# Editorial: Understanding the immuno-oncological mechanism of cancer using systems immunology approaches

**DOI:** 10.3389/fimmu.2023.1325619

**Published:** 2023-12-01

**Authors:** Rifat Hamoudi, Lucia Conti, Daniel Olive, Alexander MacRobert

**Affiliations:** ^1^ Research Institute for Medical and Health Sciences, College of Medicine, University of Sharjah, Sharjah, United Arab Emirates; ^2^ Division of Surgery and Interventional Science, University College London, London, United Kingdom; ^3^ Center for Gender-specific Medicine, Istituto Superiore di Sanità, Rome, Italy; ^4^ Faculté de Médecine, Aix Marseille Université, Marseille, France

**Keywords:** cancer, tumour immune microenvironment, immune response, systems immunology, omic analysis

Numerous studies were published in the last two decades highlighting the close relationship between host immune response and cancer progression, and immune-based therapeutic strategies hold great promise against cancer. This generated large a number of *BIG* datasets from the various OMICs platforms used to investigate the interplay between the immune system and cancer initiation and progression. In order to analyse and interpret the data generated, the field of systems immunology emerged as a novel interdisciplinary approach. It aims to integrate OMICs data generated from different high-throughput modalities and platforms using computational biology algorithms to gain a better understanding of the complex interactions and regulatory networks at the core of the interplay between the various immune cell types and cancer. Cancer is a complex chronic disease with high degree of intra-tumoral heterogeneity characterised by its adaptive ability to escape immune surveillance and acquire resistance to radio- and chemo-therapy. Systems immunology approaches elucidate some of the mechanisms involved in the complex and adaptive interplay between the tumour and immune cells that contributes to cancer progression, pathogenesis, prognosis. Systems immunology thus represents the new frontier in cancer to gain deeper understanding of the immune-cancer cell interactions to not only identify more effective and targeted therapeutic molecules and strategies but also accelerate the discovery of novel biomarkers predictive of prognosis and therapeutic response.

This Research Topic gathers different studies investigating the immune-tumour cell dynamics within the tumour microenvironment (TME) in various cancers including solid and haematological malignancies. Some of the studies proposed novel biomarkers for patient stratification and/or outcome prediction.

## Mechanisms of tumour immune microenvironment

Increasing evidence highlighted a fundamental role of tumour immune microenvironment (TIME) in cancer subtype classification and progression. Investigating such a role not only helps to elucidate the molecular mechanism of the TIME involved in cancer but it can also identify diagnostic and prognostic biomarkers for various cancers. Guo et al. introduced the immuno-score system by presenting its clinical significance and application for colorectal cancer (CRC) and highlighting the potential exploitation of this system for the screening of immunotherapy candidates. However, most of the studies describing the changes in immune response within the TIME in CRC and other cancers have focused on bulk tumour transcriptomes, with admixture of data from the tumour epithelium and stroma. Combining their data with publicly available gene expression microarray data, Shen et al. proposed a new CRC classification system based on three different immune subtypes, taking into account cancer epithelium and stroma as well as adjacent normal tissue. Furthermore, Li et al. analysed a cohort of pancreatic ductal adenocarcinoma (PDAC) patients obtained from TCGA (The Cancer Genome Atlas) database, succeeding in virtually dissecting the immune-related signatures from bulk gene expression data. They identified a new immune molecular subtype named Immune Class that may better stratify PDAC patients in cooperation with previously reported tumour and stroma classifications. Likewise, the analysis of data from TCGA database, and the use of ESTIMATE (Estimation of STromal and Immune cells in MAlignant Tumour tissues using Expression) and CIBERSORT (Cell Type Identification by Estimating Relative Subsets of RNA Transcripts) algorithms allowed Ma et al. to explore the TME and immune infiltration in high- and low-risk CRC groups. They identified a set of 13 immune related genes (IRG) that are associated to poor prognosis, and suggested the IRG-based classifier as an innovative way to predict CRC prognosis and response to immunotherapy.

Another set of studies focused on the role of infiltrating immune cell subsets in cancer progression. Hao et al. attempted to establish the prognostic value of tumour-infiltrating lymphocytes (TIL) in esophageal squamous cell carcinoma (ESCC), based on both lymphocyte subset and infiltrating location (intra- or peritumour). Specifically, they identified a positive association with both disease-free and overall (OS) survival for total TIL infiltrating the entire tumour mass, and a positive association with OS for intratumor, peritumour and total CD8+ T cells. TIL are also closely related to clinical prognosis in gastric cancer (GC). An interesting model was developed by Xie et al., who analysed gene expression profiles of GC patients, obtained from RNA-sequencing data of the GEO (Gene Expression Omnibus) database, in the context of clinical outcomes. By including four immune cell types, they constructed an immune risk score that could make an important contribution to prognosis prediction. Furthermore, starting from gene expression data of patients with gastrointestinal stromal tumour (GIST), Wang et al. observed that low T-cell infiltration correlates with high tumour purity and tumour stemness index, and identified the cancer stemness index as a new predictive biomarker of tumour metastasis in GIST patients. On the same lines, the study by Qiu et al. aimed to explore the relationship between the immune microenvironment from triple-negative breast cancer (TNBC) patient data (extracted from TCGA and GEO databases) and exosome-associated genes (downloaded from ExoBCD database) in order to construct an exosome risk model. This model was used to predict the prognosis of TNBC patients and to identify differences in immune cell infiltration rates in high- and low-risk groups.

Similar studies focused on the role of long non-coding RNAs (lncRNA) in the activation of multiple immune cells and as prognostic markers in cancer. Through the analysis of TCGA and GEO database-derived sequencing data of hepatocellular carcinoma (HC) patients, and subsequent screening of lncRNA related to survival, Nie et al. identified a nine-lncRNA risk signature that significantly correlates with immune checkpoint gene expression and TIL status. The risk score obtained from the immune-related (IR)-lncRNA signature can both predict survival of HC patients and reflect the efficacy of immune checkpoint inhibitors (ICI)-based therapy.

In addition, many studies attempted to characterise some of the molecular pathways involved in tumorigenesis. Li et al. explored the impact of the mutation status of NOTCH signalling on the prognosis of non-small cell lung cancer (NSCLC) patients on ICI-based therapy with the aim to apply immunotherapy to the greatest extent possible. They found that highly-mutated NOTCH signalling pathway is related to the inflammatory immune microenvironment and could serve as an independent predictor of NSCLC patients receiving ICI. Using imaging mass cytometry Li et al. attempted to analyse the still uncharacterised TME from subjects with untreated lung squamous cell carcinoma (LSCC). They found that CD33+ myeloid-derived cells represent the major immune suppressor cell population, and identified a novel high Foxp3/TNFα expressing nonlymphoid cell subset with proinflammatory properties. In a different cancer type, ESCC, Yu et al. determined the abundances of 22 types of immune cells by the CIBERSORT algorithm from patient gene expression data obtained from GEO and TCGA databases. They constructed a tumour-infiltrating immune cell-based prognosis signature (IPS) able to predict postoperative patient prognosis. The authors also uncovered the critical role of tumour-infiltrating M2 type macrophages in the interplay between immune status and the endothelial to mesenchymal transition phenotype in this cancer. In a similar manner but with a different approach, Hammoudeh et al. tried to reprogram the immune response through disruption of Snail-p53 binding induced by oncogenic KRAS. By analysing the transcriptome profile induced by a specific inhibitor in non-small cell lung cancer (NSCLC) cells, they reported a significant enrichment in transcripts involved in immune response and particularly those contributing to neutrophil- and T cell-mediated immunity, thus arguing to the targeting of Snail-p53 binding as a potential adjuvant immunomodulatory strategy to enhance the efficacy of current immunotherapies.

## Role of innate immune system

Most of the studies that have investigated cancer-immune cell dynamics within the TME predominantly considered the role of lymphocyte subsets in patient stratification and prognosis prediction. However, as also pointed out by Guo et al. their review, other tumour immune cell infiltrates, such as myeloid origin cells and other innate immunity cell populations, represent crucial elements of TME that strongly impact on the efficacy of anticancer therapies. Hence, the importance of a comprehensive immune classification of tumours that includes the players of innate immunity.

In this regard, Hachim et al., through the analysis of breast cancer (BC) patients obtained from TCGA database and subsequent stromal and immune profiling using ESTIMATE and CIBERSORT tools, identified and validated seven unique sub-clusters with distinct molecular and clinical profiles within the known BC subtypes. Interestingly, immune profiling analysis of these sub-clusters allowed to demonstrate a correlation of infiltrating M1 and M2 type macrophages with basal-like and luminal A-B cancer subtypes, respectively, and to associate M1 macrophage polarization markers to better prognosis. Accordingly, by performing a virtual microdissection of the bulk transcriptome at single-cell resolution, Wu et al. provided a tumour infiltrating myeloid cell landscape in lung adenocarcinoma that may help defining new immunotherapy targets. Specifically, IFIT3+ neutrophils and LAMP3+ dendritic cells were related to responsiveness or unresponsiveness to immune-targeted therapy, respectively, whereas the infiltration levels of TIMP1+ macrophages and S100A8+ neutrophils were both significantly associated with poor prognosis.

Remaining on the role of innate immunity and accessory stroma cells, the study by Yin et al. reported the generation of a dynamic transcriptome map of different TME cell types during GC progression using single-cell sequencing analysis. They found a set of key transition markers related to tumour evolution and delineated landmark dynamic carcinogenic trajectories of these cells, suggesting a phenotypic convergence of different TME cell types (macrophages, fibroblasts, endothelial cells) toward tumour generation processes. Along the same line, unsupervised hierarchical clustering was used by Køstner et al. to explore the association of distinct immune phenotypes within the tumour with the level of systemic inflammation in resectable colon cancer patients. They found that tumour-associated systemic inflammation correlates with a myeloid-dominated TME and suggested a role for C-reactive protein as an informative biomarker of the immune response taking place at the tumour site.

Another innate immunity focused study by Cianga et al. addressed NK cell maturation in the pre-leukemic state of acute myeloblastic leukemia and during leukemic transformation. Unlike previous work that had focused on peripheral NK cells, they performed unsupervised analysis of NK cell subsets from bone marrow aspirates identifying a shift from the mature toward the immature state and an impaired NK cell-mediated antitumor response during cancer progression.

Clear cell renal cell carcinoma (ccRCC) is a highly infiltrated tumour with different types of immune cells having differential effects on patient prognosis. Zhang et al. systematically analysed chromatin accessibility, whose changes have been associated with tumour initiation, migration and metastatic progression, in two clusters of ccRCC patients having differentially immune-infiltrated cells and different prognosis. They found that the differential peaks and prognosis-related immune signal cells are similarly distributed in the chromosomes and that key transcription factors may play an important role in the two different immunological subtypes.

## Role of systemic immune response

Another important aspect of cancer immunology is the role of systemic immune and inflammatory responses that may mirror the immune dynamics in the tumour environment and whose dissection may help identify new and more easily detectable biomarkers predictive of prognosis or therapy response. In the context of many malignancies there is evidence that the transcriptome of peripheral blood immune cells is altered. In this regard, Moradpoor et al. have demonstrated that the protein expression profile in PBMC from BC patients strictly reflects the patterns of proteins expressed in the tumour tissue and have identified and validated a series of PBMC-associated biomarkers that match those previously reported in metastatic BC. Moreover, in their study Ding et al. discussed the role that soluble PD-L1, which is associated with prognosis in many malignancies, might play in predicting the outcome in glioma patients receiving radiotherapy (RT). The baseline level of circulating soluble PD-L1 indeed was found to correlate with poor prognosis and its increase after RT suggests that the strategy of combining ICI-based therapy and RT might be promising for glioma treatment. The T cell receptor (TCR) repertoire of tumour tissue or peripheral blood represents an indicator of prognosis in subjects with various types of cancer, and changes in the peripheral blood TCR repertoire may be used to monitor the body response to immunotherapy. Wang et al. reported the results of TCRβ CDR3 profiling performed by high-throughput sequencing in matched tumour tissue, regional lymph nodes and peripheral blood of subjects with papillary thyroid carcinoma. These data are made available as a reference for further studies on the immunological mechanisms in thyroid carcinoma.

## Immune response to cancer therapy

Immunotherapy has been shown to work effectively in some cancers but not others. Many studies investigated the current immunotherapies and the need of more effective and highly cancer-specific immune-based strategies as well as reliable biomarkers for personalised therapy. In particular, Kubo et al. highlighted the low response to ICI-based therapy in the majority of cancer types and the immune-related adverse effects, whereas Kozani et al. discussed the state of the art of chimeric antigen receptor (CAR) T cell therapy in solid tumours and the role of suppressive TME in determining negative responses. Bracci et al. provided a summary of recent studies in which multi-omics technologies have been used to characterize the mechanisms of response and to identify powerful biomarkers of response to ICI, CAR-T cell therapy, dendritic cell- and peptide-based cancer vaccines. They also give an overview of the current high-throughput methodologies suitable for systems immunology as well as of the key steps of data integration and biological interpretation.

Additionally, the targeting of CTLA4 and PD1/PDL1 has shown promise as immunotherapy for some cancers, however, many patients fail to respond. Lymphocyte activating 3 (LAG3) is involved in the negative regulation of lymphocyte-antigen presenting cell interaction and may serve as an alternative inhibitory receptor to be targeted in the clinic. Liu et al. investigated transcriptome data and associated clinical information derived from almost 3000 BC patients demonstrating a role for this molecule as a potential biomarker. Furthermore, it was shown that it can synergise with CTLA4, PD1/PDL1 and other immune checkpoints, thereby contributing to improved combination immunotherapy. CKLF-like MARVEL transmembrane domain-containing 6 (CMTM6) has been reported to stabilize PD-L1 and to enhance the efficacy of immunotherapy. By analysing data from TCGA and from the CPA (Cancer Proteome Atlas) database for 32 cancer types, Zhao et al. confirmed the important role played by CMTM6 in TME and highlighted its potential as prognostic biomarker in some types of cancers and as a target for cancer immunotherapy. Narducci et al. reported on a patient simultaneously affected by synchronous metastatic melanoma and Sezary Syndrome (SS), an aggressive variant of cutaneous T cell lymphoma. The authors reported a significant clinical and biological response to SS in the patient under PD-1 blocking therapy for melanoma and suggested that the changes in immune cell phenotype and frequency they observed during therapy could help determine early SS patient’s clinical response.

Photodynamic therapy (PDT) is form of targeted therapy that has been used to treat various cancers including prostate, breast, and skin. It involves focal illumination with visible or near-Infrared light of the diseased lesion following systemic or topical administration of a photosensitising agent. To enhance tumour targeting, the photosensitiser can also be tagged with a specific cancer biomarker antibody such as HER2, which has given rise to the term ‘photoimmunotherapy’. The interaction of light with the photosensitiser leads to generation of cytotoxic reactive oxygen species, which attack multiple intracellular targets. Banerjee et al. used PDT to treat triple negative breast cancer (TNBC). They showed that verteporfin-PDT with 5-aza-2’deoxycytidine is effective in treating an orthotopic TNBC murine model via the induction of specific immune response biomarkers including various chemokines such as CCL2, CCL4 and CCL5, CD4 and CD8 T-cell response, and innate immune biomarkers such as Granzyme A (GZMA) and Perforin 1 (PRF1) as well as inflammatory biomarkers related to the NF-κB pathway such as Bcl3. The study showed the importance of the interplay of various immune cells in the therapeutic effect of PDT.

## The role of systems immunology in immuno-oncology

In the collected studies, the high number of patients’ data extracted from known cancer databases such as TCGA and GEO, and the mathematical approaches such as unsupervised learning, Bayesian calculus, maximum likelihood estimation, and predictive algorithms, and computational methods including GSEA, subclass mapping and connectivity map analysis applied, shed light on the interactions within cellular and molecular networks of the immune system in the various cancer models investigated. The use of systems immunology approaches thus provided insights beyond the isolated immune component (cell or function) that is analysed using *ex vivo* or *in vivo* studies, as well as the prediction of the overall immune functions and immune-cancer cell interactions. Taking advantage of this novel approach, the studies in this Research Topic identified novel biomarkers for cancer patients’ stratification and prognosis prediction, which were validated on independent patients’ cohorts.

## Conclusions and thoughts

Cancer is a heterogenous complex chronic disease, and the immune system is designed to adapt and maintain homeostasis (steady state) through the containment of environment or genetic perturbations that eventually lead to cancer. Understanding the relationship between two complex adaptive systems requires the generation of large amount of data from different modalities that examine the interplay between cancer and immunology from different perspectives. Systems immunology is an emerging field that combines biology, immunology, bioinformatics and mathematics to understand complex biological systems at the molecular level.

This Research Topic showed that investigating the immunological changes that result from cancer using OMICs, as well as other molecular data, can lead to the identification of more homogeneous cancer subgroups and biomarkers with more accurate prognostic and predictive value. This may lead to more accurate diagnosis and prognosis of various cancers at the early stages paving the road for more effective and personalized therapeutic approaches. In addition, studying the associated immunological response to cancers may shed light on the complex molecular mechanisms involved in cancer progression and metastasis as well as in resistance to therapy.

In future, the advent of Artificial Intelligence and Machine Learning as well as the development of more sophisticated mathematical algorithms may further improve the multidisciplinary approaches and tools to better characterize the immuno-oncology system crosstalk leading to better understanding of the cancer initiation, progression and metastasis thereby providing more effective therapy to the patients.

## Author contributions

RH: Conceptualization, Data curation, Formal Analysis, Methodology, Project administration, Supervision, Validation, Writing – original draft, Writing – review & editing. LC: Conceptualization, Data curation, Formal Analysis, Methodology, Resources, Validation, Writing – original draft, Writing – review & editing. DO: Conceptualization, Data curation, Formal Analysis, Methodology, Writing – review & editing. AM: Data curation, Formal Analysis, Methodology, Writing – review & editing.

